# A multimodal dataset of real world mobility activities in Parkinson’s disease

**DOI:** 10.1038/s41597-023-02663-5

**Published:** 2023-12-20

**Authors:** Catherine Morgan, Emma L. Tonkin, Alessandro Masullo, Ferdian Jovan, Arindam Sikdar, Pushpajit Khaire, Majid Mirmehdi, Ryan McConville, Gregory J. L. Tourte, Alan Whone, Ian Craddock

**Affiliations:** 1 Movement Disorders Group, Bristol Brain Centre, North Bristol NHS Trust, Southmead Hospital, Southmead Road, Bristol, BS10 5NB UK; 2 Translational Health Sciences, University of Bristol, 5 Tyndall Ave, Bristol, BS8 1UD UK; 3 Faculty of Engineering, University of Bristol, Digital Health Offices, 1 Cathedral Square, Bristol, BS1 5DD UK; 4 School of Natural and Computing Sciences, University of Aberdeen, Aberdeen, UK; 5 Edge Hill University, Ormskirk, UK; 6 Datta Meghe Institute of Higher Education and Research, Wardha, India; 7 Advanced Research Computing, University of Oxford, Oxford, UK

**Keywords:** Electrical and electronic engineering, Parkinson's disease, Outcomes research

## Abstract

Parkinson’s disease (PD) is a neurodegenerative disorder characterised by motor symptoms such as gait dysfunction and postural instability. Technological tools to continuously monitor outcomes could capture the hour-by-hour symptom fluctuations of PD. Development of such tools is hampered by the lack of labelled datasets from home settings. To this end, we propose REMAP (REal-world Mobility Activities in Parkinson’s disease), a human rater-labelled dataset collected in a home-like setting. It includes people with and without PD doing sit-to-stand transitions and turns in gait. These discrete activities are captured from periods of free-living (unobserved, unstructured) and during clinical assessments. The PD participants withheld their dopaminergic medications for a time (causing increased symptoms), so their activities are labelled as being “on” or “off” medications. Accelerometry from wrist-worn wearables and skeleton pose video data is included. We present an open dataset, where the data is coarsened to reduce re-identifiability, and a controlled dataset available on application which contains more refined data. A use-case for the data to estimate sit-to-stand speed and duration is illustrated.

## Background & Summary

Parkinson’s disease (PD) is a slowly-progressive neurodegenerative disorder, characterised by symptoms such as slowness of movement and gait dysfunction^1^ which fluctuate across each day^2^. Currently, PD management relies on therapies which improve symptoms. Despite there being multiple putative disease-modifying treatments (DMTs) showing promise in laboratory studies^3^, there is no licensed treatment which has been demonstrated to change the course of the underlying disease. One reason for this is the dearth of sensitive, frequent, objective biomarkers to enhance the current gold-standard clinical rating scale^4^ used to measure disease progression of PD. This scale, the Movement Disorders Society-sponsored revision of the Unified Parkinson’s Disease Rating Scale (MDS-UPDRS), has limitations including its “snapshot” nature, which cannot fully capture the symptom fluctuations experienced by patients, its non-linear and discontinuous scoring system, inter-rater variability^5^ and the impact of the Hawthorne effect^6^ (how being observed changes a person’s behaviour)^7,8^ on symptoms.

This dataset was created as a basis on which to build approaches which may overcome flaws relating to symptom quantification in PD clinical trials. The aim was to use cameras and wearables to measure PD symptoms and activities from unrestricted “free” living in a home setting, so that potential digital biomarker(s) of disease progression could be identified that could be continuously, passively and unobtrusively quantified by a scalable sensor platform. Free living behaviour (living as naturally as possible with very few external interventions) was captured because it has been shown that mobility symptoms in PD change during assessments in a clinical setting^8^. From this data, a paper was produced evaluating turning of gait, which showed that mobility outcomes also changed when the participants are observed during clinical assessments in a home setting^9^.

### Related work

Other PD symptoms that have been evaluated in home settings using technology, especially with body-worn wearable devices and smartphone sensors^10^, include motor symptom fluctuations^11^, tremor, bradykinesia and dyskinesia^12,13^, activity levels^14^ and sleep^15^.

The importance of turning in gait, which combines straight-ahead gait ability and postural stability, as a proxy metric to track disease progression in PD, is increasingly recognised^16^. Gait and turning can be remotely quantified using a single wearable device which can passively capture the activity^12,17,18^. However, a gap remains in the demonstration of the accuracy of devices to measure these outcomes from real-world data; the work of the Mobilise-D consortium, currently underway, aims to address this evidence gap^19^. Gait is perhaps more amenable to quantification by home-based sensing systems than more subtle symptoms such as fine finger motor function, which may be lost to detection in the “noise” of real-world daily life. Much existing work evaluating gait in home settings has used wearable sensors containing inertial motor units^12,18^, with sensors often applied to the back or lower limbs. The wrist is considered more acceptable by participants than other body sites for long-term wearable sensor placement in PD^20^, but potential changes in arm swing^21^ and upper limb tremor^22^ need to be accounted for in gait analysis. Single or multiple cameras can also be used in real-world settings^23^ to measure symptoms and activities. Cameras capture a broader spectrum of information about activities than wearables. The use of cameras in addition to wearable sesnsors increases the breadth of activities that can be covered. However, each camera has limited coverage, and therefore a larger number of cameras increases the number of distinct activities captured in daily living^24^. Many of the works looking at camera data have focussed on participant performance in evaluation of structured clinical rating scales during telemedicine consultations as opposed to naturalistic behaviour^25^. Cameras can detect Parkinsonian gait and measure some gait features including step length and average walking speed^26,27^. Time of flight devices (which measure distances between the subject and the camera) have been used to assess medication adherence through gait analysis^28^. Also, multimodal data fusion of in-home camera and wearable data shows promise for privacy-preserving tracking of Parkinsonian symptoms^29,30^.

In the literature, there are many datasets available for human activity and action recognition, some of which are detailed in Table 1. These explore a wide variety of activities such as walking, poses, gestures, and everyday tasks such as eating or cleaning; some examples are given in Table 2. A subset of these datasets also explores “transitions”, which is to say, moving from one pose or activity to another, such as moving from a seated to standing position. As can be seen from Table 1, the majority of these datasets present data from relatively young and healthy participants. For PD action recognition, the MPower dataset^31^, published in 2016, collected scripted activities including walking and turning when prompted by the app from more than 3000 people, both with PD and without. There was no ground truth labelling of this IMU data and the activities collected were scripted. There is a paucity of other sizeable and good-quality motion datasets at present that involves participants with PD. Machine learning approaches such as deep convolutional neural network models trained on healthy populations show reduced accuracies when tested on populations of people with PD^32^, so PD-specific datasets for model training are ideally needed. This is especially important since dopaminergic medication^33^, the severity of motor symptoms such as bradykinesia^34^ and clinical phenotype (tremor-dominant or postural instability and gait difficulty)^35^ all influence gait dysfunction in PD.

**Table 1:** A selection of benchmark datasets currently available for comparison with PD SENSORS dataset.

Dataset	Participants	Sensor					Data					
		Depth	RGB	MoCap	Inertial	Synth	Silhouette	Depth	2D Skel	3D skel	RGB	Inertial
CMU MoBo^87,88^ (2001)	25								Outline		×	
Casia^89^ (2005)	> 1000		×				×				×	
HDM-05^90,91^ (2005)	5 actors			×					24-joint		×	
HumanEva-I^92^ (2006)	4		×	×						15-joint	×	
HumanEva-II^92^ (2006)			×	×						15-joint	×	
MSRAction3D^93^ (2010)	10	×						×				
RGBD-HuDaAct^94^ (2011)	30 students	×	×					×			×	
MSRDailyActivity3D^95^ (2012)	10	×	×					×		20-joint	×	
HMDB-51^96^ (2011)	YT (YouTube), movies		×								×	
UCF101^97^ (2012)	YT		×								×	
HUMAN 3.6 M^98,99^ (2014)	11 actors			×			×	×		32-joint	×	
MPI-INF-3DHP^100^ (2017)	8 actors		×							17/21-joint		
OU-isr gait DB^101^ (2017)	63,846 (2–90 years)		×				×				×	
JTA^102^ (2018)	Simulated					×				22-joint	×	
DU-MD^103,104^ (2018)	50 (16-22 years)				×							×
KIMORE^105^ (2019)	78, 34 w/motor dysfunction	×	×					×		25-joint	×	
Up-Fall^106^ (2019)	17 (18–24 years)				×							×
FallAllD^107^ (2020)	15				×							×
VISTA^108^ (2022)	20 (19–44 years)	×	×		×			×	25-joint		×	×
Sphere Challenge (2023)	10 (18–39 years)	×	×		×							×

Joint count is indicative, as dataset reporting of this information varies. RGB: red-green-blue video; MoCap: motion capture; Synth: synthetic; 2D: 2-dimensional; Skel: skeleton pose; 3D: 3-dimensional.

**Table 2:** Selected activities and transitions labelled in benchmark human activity recognition datasets.

Dataset	Transitions					Activities								
	Sit-to-stand	Turn	Lie down/get up	Fall	Ascend/descend stairs	Walk	Jog	Loaded-walk	Gesture	Punch/hit	Throw/catch	Eat/drink	Clean/cook	Hygiene/dressing
CMU MoBo^87,88^ (2001)						×		×						
Casia^89^ (2005)						×		×						
HDM-05^90^ (2005)						×								
HumanEva-I^92^ (2006)						×	×		×	×	×			
HumanEva-II^92^ (2006)						×	×		×	×	×			
MSRAction3D^93^ (2010)							×		×	×	×			
RGBD-HuDaAct^94^ (2011)	×		×									×	×	×
MSRDailyActivity3D^95^ (2012)	×		×			×					×	×		
HMDB-51^96^ (2011)	×	×		×	×	×			×	×	×	×		
UCF101^97^ (2012)								×					×	×
HUMAN 3.6 M^98,99^ (2014)						×		×				×		
MPI-INF-3DHP^100^ (2017)						×		×	×			×		
OU-isr gait DB^101^ (2017)						×								
JTA^102^ (2018)						×	×					×		
DU-MD^104^ (2018)				×	×	×	×							
KIMORE^105^ (2019)		×												
Up-Fall^106^ (2019)				×		×								
FallAllD^107^ (2020)	×		×	×		×	×		×					
VISTA^108^ (2022)						×						×	×	×
Sphere Challenge (2023)	×		×		×	×		×						

The difficulty in validating the sensor data without any “ground truth” (a definite knowledge of what the person is actually doing) is a well-acknowledged challenge in the academic field of identifying and evaluating PD outcome measures^17,36^. For instance, data collected from unwitnessed real-world living provides challenges in interpretation related to sources of variability. In PD, gait is influenced by multiple such confounding variables, such as cognitive impairment^37^, depression^38^ and the use of mobility aids^39^. Conducting another activity (like holding a phone) while simultaneously mobilising, or “dual tasking”, influences both sit-to-stand^40^ and turning in gait^9,41^. Therefore, it is valuable for such significant sources of variability to be either minimised or carefully characterised in datasets intended to evaluate human gait.

Furthermore, validation of algorithms to measure real-world gait in PD ideally requires a ground truth dataset from a real-world setting since a body of literature has demonstrated that such algorithms developed in the laboratory translate poorly to naturalistic environments^42–48^.

### Our dataset and potential uses

Here, we present REMAP (REal-world Mobility Activities in Parkinson’s disease), a unique, labelled real-world dataset of mobility-related activities in people with PD and healthy control volunteers. The activities are manually labelled by human raters, contain both “on” and “off” medication states in the PD participants (as defined in the Methods section: ‘‘On’’ and ‘‘off’’ medication), and capture both free unscripted living and observed clinical assessments in a home setting.

Beyond identifying mobility activities, a variety of extra annotations are provided that add rich information about the actions, shown in Table 3. We include pseudonymised and coarsened skeleton pose data in an open source dataset^49^, and additional data including accelerometry and uncoarsened skeleton pose data is published in a controlled dataset^50^, which is available on an application basis to researchers. Sources of variability (e.g. walking aids, cognitive impairment, depression) are minimised by the study’s exclusion criteria. It is our hope that this dataset may be instrumental in fostering the improvement of computational models to evaluate mobility outcomes in PD. The difference between observed and unobserved, free-living and clinical assessment, and medication-related mobility outcomes may be explored. Vitally, this dataset could bridge the proven gap between laboratory and home-like setting algorithm validation. To the authors’ knowledge, it is the first human-labelled real-world dataset using ground truth from static cameras in PD.

**Table 3:** Outline of human rater label dataset contents.

Activity labelled	Parameters included for each label
Turning of gait	Turning duration (seconds:milliseconds)
	Angle of turn to nearest 45 degree (degrees)
	Number of turning steps (integers)
	Type of turn (pivot or step)
	PD or control status
	“On” or “off” medication status (for PD participants)
	“On” or “off” deep brain stimulation status (for PD participants)
	Clinical assessment (“Yes” or “No”, with “No” therefore denoting free-living behaviour)
Sit-to-stand	Whole episode duration (seconds:milliseconds)
	Final attempt duration (seconds:milliseconds)
	Extra detail about STS transition: uses flat surface(s) to push off from arms of chair, >1 attempt, moves forward in chair, carrying something in hand(s)
	MDS-UPDRS question 3.9 rating (on 0–4 scale)
	PD or control status
	"On" or "off" medication status (for PD participants)
	“On” or “off” deep brain stimulation status (for PD participants)
	Clinical assessment (“Yes” or “No”, with “No” therefore denoting free-living behaviour)
Non-turning, non-sit-to-stand	Action labelled (Descending stairs, sitting etc)
	Episode duration (seconds:milliseconds)
	PD or control status
	“On” or “off” medication status (for PD participants)
	“On” or “off” deep brain stimulation status (for PD participants)
	Clinical assessment (“Yes” or “No”, with “No” therefore denoting free-living behaviour)

## Methods

### Participants

24 participants were recruited to this study, 12 with PD (mean age 61.25; 7 males, 5 females) and 12 healthy control volunteers (mean age 59.25, 3 males, 9 females). Participants’ cohort-level demographic data is presented in Table 4. Participants with PD were recruited through movement disorders specialist clinics or general neurology outpatient clinics in North Bristol NHS Trust, through posters in the outpatient department of North Bristol NHS Trust, via Cure Parkinson’s (UK-based charity), a local Patient and Public Involvement Group and by word-of-mouth. The medical care for the participants with PD continued unchanged. The control participants recruited were a friend or family member of the PD participants. They volunteered their contact details to the research team through their study partner. They were offered a separate consultation with the research team to ensure independent and informed consent. Most of the participant pairs were spouse-spouse pairings, but there were also parent-child and friend-friend pairs. Inclusion criteria for the study for PD participants included:

- Diagnosis of idiopathic PD according to UK Brain Bank Criteria^51^.
- Age over 18.
- Modified Hoehn and Yahr Scale score^52^ of 3 or less in “off” state (i.e. when the patient’s symptoms are greater as they are withholding their symptom-improving dopaminergic medications).

**Table 4:** Cohort-level demographic and clinical rating tool scores of all participants.

	Parkinson’s disease participants	Control participants
	Mean (standard deviation)	Mean (standard deviation)
Age (years)	61.25 (8.5)	59.25 (13.4)
Number of men (%)	7 (58)	3 (25)
MDS-UPDRS total score	“On” = 44.8 (16.1); “off = 61.7 (29.9)	6.8 (4.8)
MDS-UPDRS III sub score	“On” = 19.1 (10.4); “off” = 36.8 (23.0)	2.8 (1.9)
PIGD sub score	“On” = 2.8 (1.9); “off” = 4.3 (4.8)	0.1 (0.3)
TUG-test time	“On” = 8.3 (2.1); “off” = 10.8 (5.1)	7.0 (1.5)
PDQ-39	23.3 (14.2)	
RBD-SQ	6.8 (3.3)	
PDSS	118.9 (14.2)	
NMSS	31.8 (17.4)	
Hoehn and Yahr “off” medications	2.3 (0.8)	
Years since diagnosis	8.2 (6.5)	
LEDD	517.5 (395.7)	

MDS-UPDRS: Movement Disorder Society sponsored revision of the Unified Parkinson’s Disease Rating Scale; PIGD: Postural Instability and Gait Difficulties sub-score of MDS-UPDRS; TUG-test: Timed-Up-and-Go test; PDQ-39: Parkinson’s Disease Questionnaire-39; RBD-SQ: REM sleep Behaviour Disorder Screening Questionnaire; PDSS: Parkinson’s Disease Sleep Scale; NMSS: Non-Motor Symptoms Scale for Parkinson’s disease; LEDD: levodopa equivalent daily dose.

Exclusion criteria for the study included:

- Current significant depression or cognitive impairment.
- Use of walking aids whilst inside the house to aid mobility either “on” or “off” medications.

Exclusion criteria specifically for control participants:

- History of PD, REM (rapid eye movement) sleep behaviour disorder, dementia, or other neurodegenerative/significant musculoskeletal condition.

**Fig. 1:**
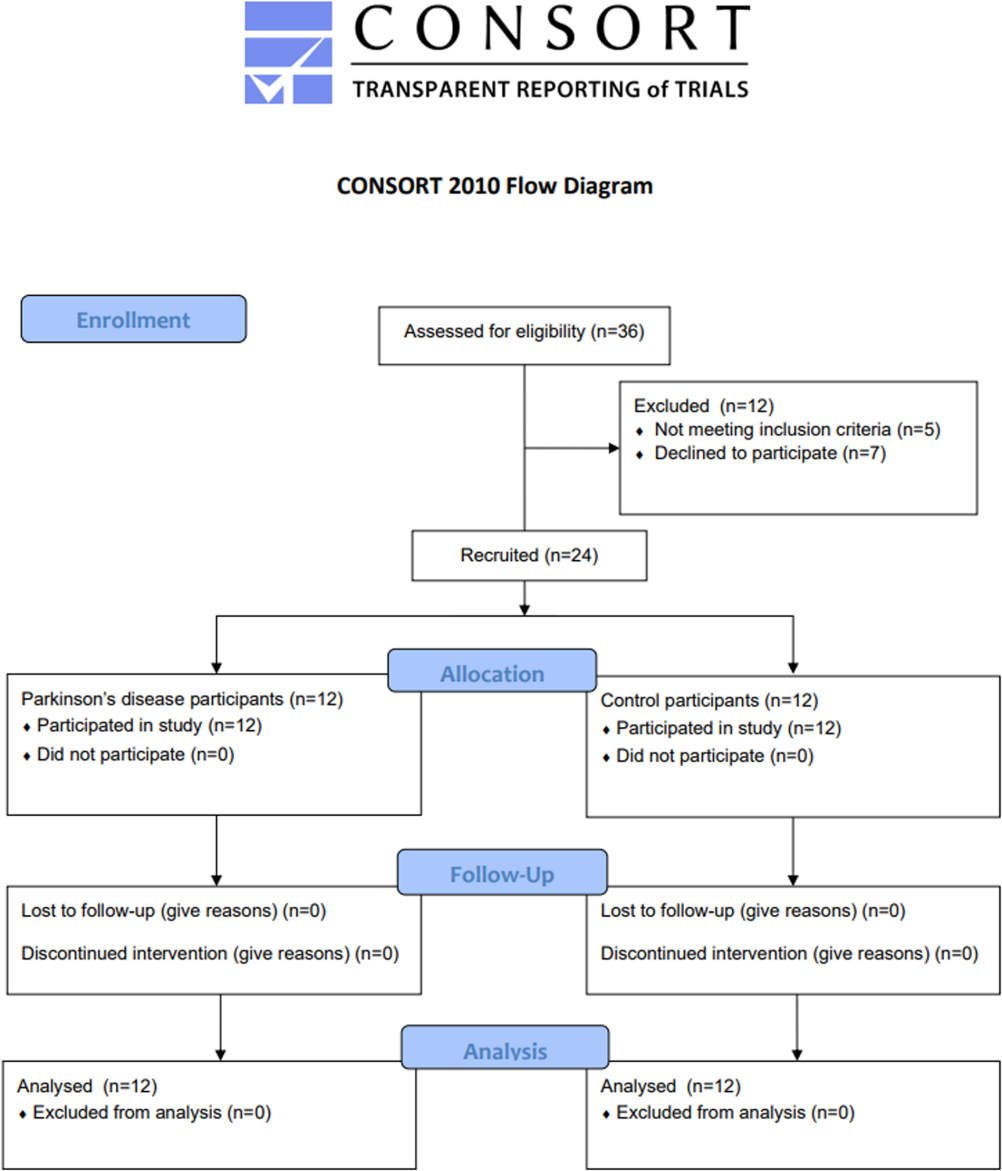
CONSORT diagram illustrating study recruitment and drop-out.

The recruitment and drop-out data are shown in Fig. 1.

### Consent and ethics

Written informed consent was gained from all study participants, including permission to publish anonymised data openly and to share pseudonymised data with other researchers or academic third parties, in scientific meetings and through paper and electronic publications. Full approval from NHS Wales Research Ethics Committee 6 was granted on 17th December 2019, and Health Research Authority and Health and Care Research Wales approval confirmed on 14th January 2020; the research was conducted in accord with the Helsinki Declaration of 1975. The data was pseudonymised with a unique identification number (ID) assigned to each participant for the study; new randomly-generated unique ID numbers were then assigned for the purposes of this dataset publication.

### Study protocol

#### Setting

**Fig. 2:**
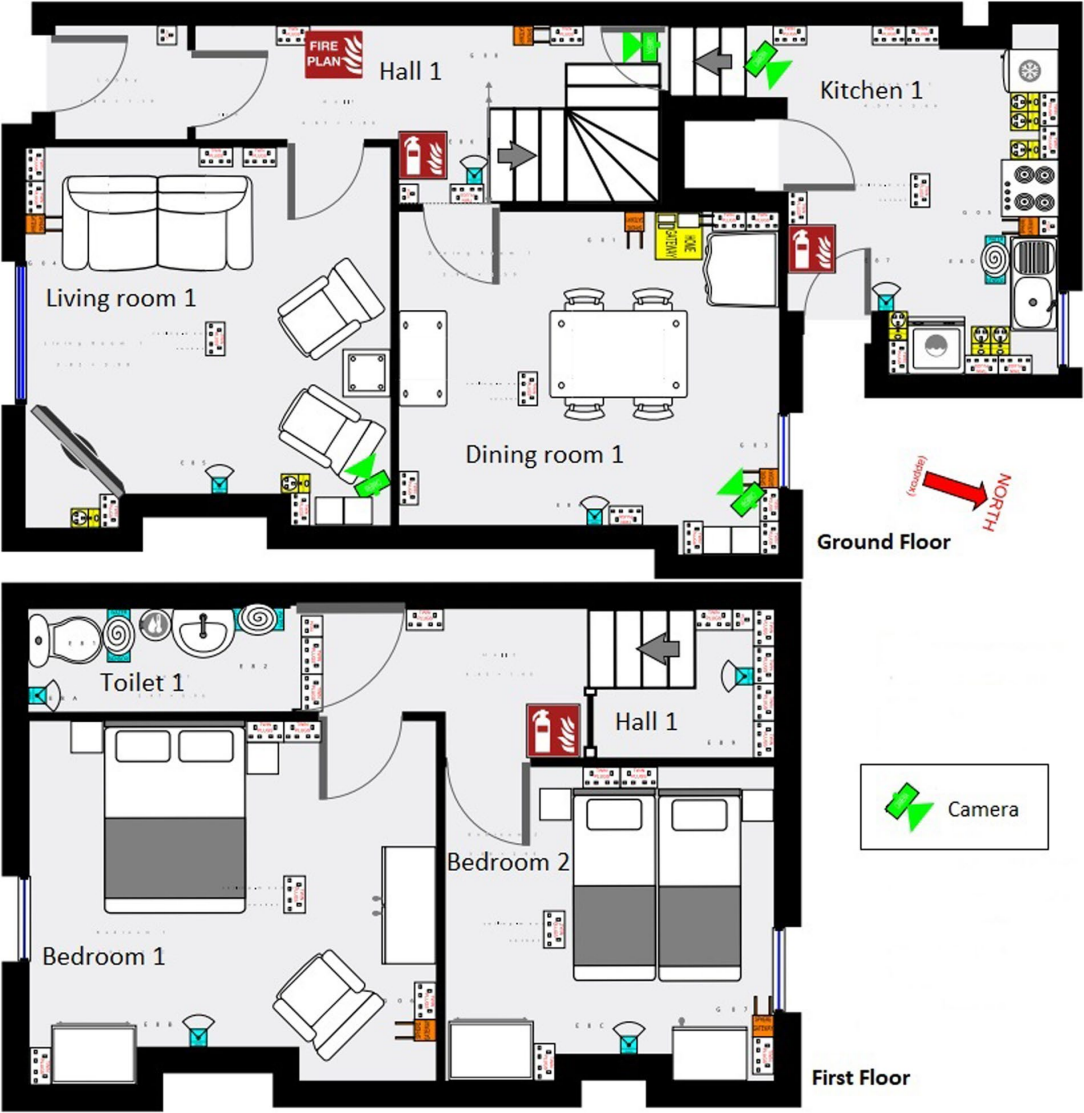
Layout of test-bed house used for data collection.

The participants stayed for 5 days, 4 nights continuously at a fully-furnished 2-bedroom terraced house which was the study setting. There were wall-mounted cameras in communal rooms downstairs, as shown in Fig. 2.

#### Free-living

Apart from the clinical assessment testing sessions described in Clinical assessments, the participants were encouraged to live as freely as possible, without external intrusion. They could take part in activities outside the test-bed house; however they were asked to stay overnight when possible. They could continue working if they needed and bring anything to the house that helped them continue their normal hobbies e.g. exercise DVDs. While the aim was to facilitate naturalistic behaviour, this was not the participants’ home and they were informed when video data was captured. These two factors may have influenced their behaviour subtly, as explored in Morgan *et al*. in their related qualitative work^53^. However, the strong feedback provided by participants was that the participants felt their behaviour overall was naturalistic. Our group have also shown that the turning outcomes captured in this dataset do alter between the researcher-observed clinical assessments and unobserved free-living^9^ - this demonstrates the potential of the dataset to reveal the impact of the Hawthorne effect on mobility outcomes.

#### “On” and “off” medication

The person with PD in each study pair was asked to withhold their dopaminergic medications (12 hours for short-acting levodopa-containing agents and 24 hours for long-acting therapies) and/or switch off their deep brain stimulators (DBS) so that they were in the practically-defined “off” medications/DBS state for a limited period of hours. Therefore, when sit-to-stand (STS) or turning episodes are labelled as “on” medications/DBS, they took place during the rest of the study, before or after the practically-defined “off” state.

#### Clinical assessments

Each participant underwent multiple clinical evaluations (on 2 occasions for the control participants, 3 for the participants with PD). The first occasion was on the first day of the 5-day stay in the testbed house. The other occasion(s) were on the day when the participant with PD withheld their medications. For logistic purposes, for 10 pairs of participants this testing was on day 4 of the study, but in one case it was day 3 and for one case it was day 5. The clinical assessments lasted between 30 minutes and an hour, and consisted of performing the full motor sub score of the MDS-UPDRS (III)^4^ and the timed-up-and-go (TUG) test^54^ twice. Participants were also asked to do 20-metre walks as part of their clinical assessment, during which the clinician evaluated their gait. Each continuous 20-metre walk included 3 × 180 turns. At each testing session they completed the 20-metre walk 3 times, each at a different pace: “normal”, “fast” and “slow”. The participants with PD were also asked to complete some patient-reported outcome measures which evaluate other aspects of living with PD: the Parkinson’s Disease Questionnaire-39^55^, the Parkinson’s Disease Sleep Scale-2^56^, the Non-Motor Symptoms Scale^57^ and the Rapid Eye Movement-sleep Behavior Disorder Screening Questionnaire^58^.

The MDS-UPDRS is a clinical rating scale widely used in clinical trials to evaluate symptoms and their progression. It consists of four sub-scales, of which part III is the direct clinical evaluation of PD motor symptoms by a clinician. There are 33 questions in part III, each scored on a 0–4 scale (e.g. where 0 is no symptoms and 4 is very severe symptoms). The MDS-UPDRS part III comprises diverse symptoms including tremor, facial expression, slowness of various movements, rigidity and gait. For this study, the evaluation of turning in gait during clinical assessments takes place when the participants do the TUG test and the 20-metre walk. STS is evaluated during the MDS-UPDRS III (the participant crosses their hands in front of their chest and, using an upright chair, stands from sitting). The maximum possible total score for the MDS-UPDRS III sub scale is 132. A sub-score within the MDS-UPDRS is the Postural Instability and Gait Difficulties score (PIGD), which is comprised of parts 2.12, 2.13, 3.10, 3.11 and 3.12^59^. It looks to identify self-report and objective performance in axial stability and gait from within the wider tool.

The TUG test is a simple assessment of functional mobility which involves the person arising from a seated position to walk forward 3 metres, turn 180° and walk back to sit down in the chair as fast as they feel able^54^. It shows good to excellent intra-rater, inter-rater and test-retest reliability for total duration and turning duration in PD^60,61^.

The Parkinson’s Disease Questionnaire-39^55^ is a 39-question tool to evaluate self-report of health-related quality of life. The Parkinson’s Disease Sleep Scale-2 is a 15-point visual analogue scale that has been validated in PD^56^ and allows patients with PD to self-rate the profile of nocturnal disturbances and sleep disruption they experience. The Non-Motor Symptoms Scale is a 30-question tool which assesses the severity and frequency with which a patient with PD experiences a variety of non-movement related symptoms, such as urinary dysfunction and mood/cognitive symptoms^57^. The Rapid Eye Movement (REM) sleep Behaviour Disorder Screening Questionnaire is a useful screening tool due to its relatively high sensitivity for REM sleep behaviour disorder^58^, which affects a significant proportion of patients with PD.

#### Video data capture

**Fig. 3:**
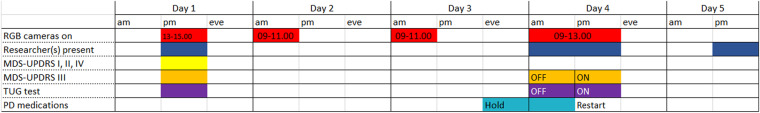
Example of a typical study schedule across five days for participants. RGB: red-green-blue video data; MDS-UPDRS: Movement Disorder Society sponsored revisin of the Unified Parkinson’s Disease Rating Scale; TUG-test: Timed-Up-and-Go test; PD medications: Parkinson’s disease medications.

Red-green-blue (RGB) video was captured from the cameras shown in Fig. 2 for between 1 and 3 hours on the first 4 days of the study for each pair. The timings were chosen a) to capture the clinical evaluation episodes and b) to capture the participants free-living (without external influence or scripts) when they were alone in the house. The free-living timings were prearranged with the participants to ensure that they were likely to be at home while the data was collected. Typical RGB capture times and their relation to the other study activities are illustrated in Fig. 3. A total of 85 hours of study time was recorded by the RGB cameras (averaging 1.8 hours per day per participant pair), usually by all cameras collecting data simultaneously. All turning and STS episodes, along with other activities such as sitting and standing still, were labelled using this RGB data.

### Sensor specifications

#### Cameras

Off-the-shelf Microsoft Kinect cameras were wall-mounted above eye level, placed to achieve maximum coverage of the room they were in and to minimise occlusions. They were situated in communal rooms downstairs, shown in Fig. 2, including the hallway, dining room, living room and kitchen. While these cameras are capable of generating both RGB and depth images, for the purposes of this study, RGB video was collected.

#### RGB data

RGB data frames from the Kinect were stored alongside corresponding timestamps, then aggregated into an MPEG-4 (Moving Pictures Expert Group 4)-encoded video file according to the timestamp. The resulting video from these platforms was 640 × 480 pixels in resolution and had an average frame rate of 30 frames per second.

#### 2D skeleton data

**Fig. 4:**
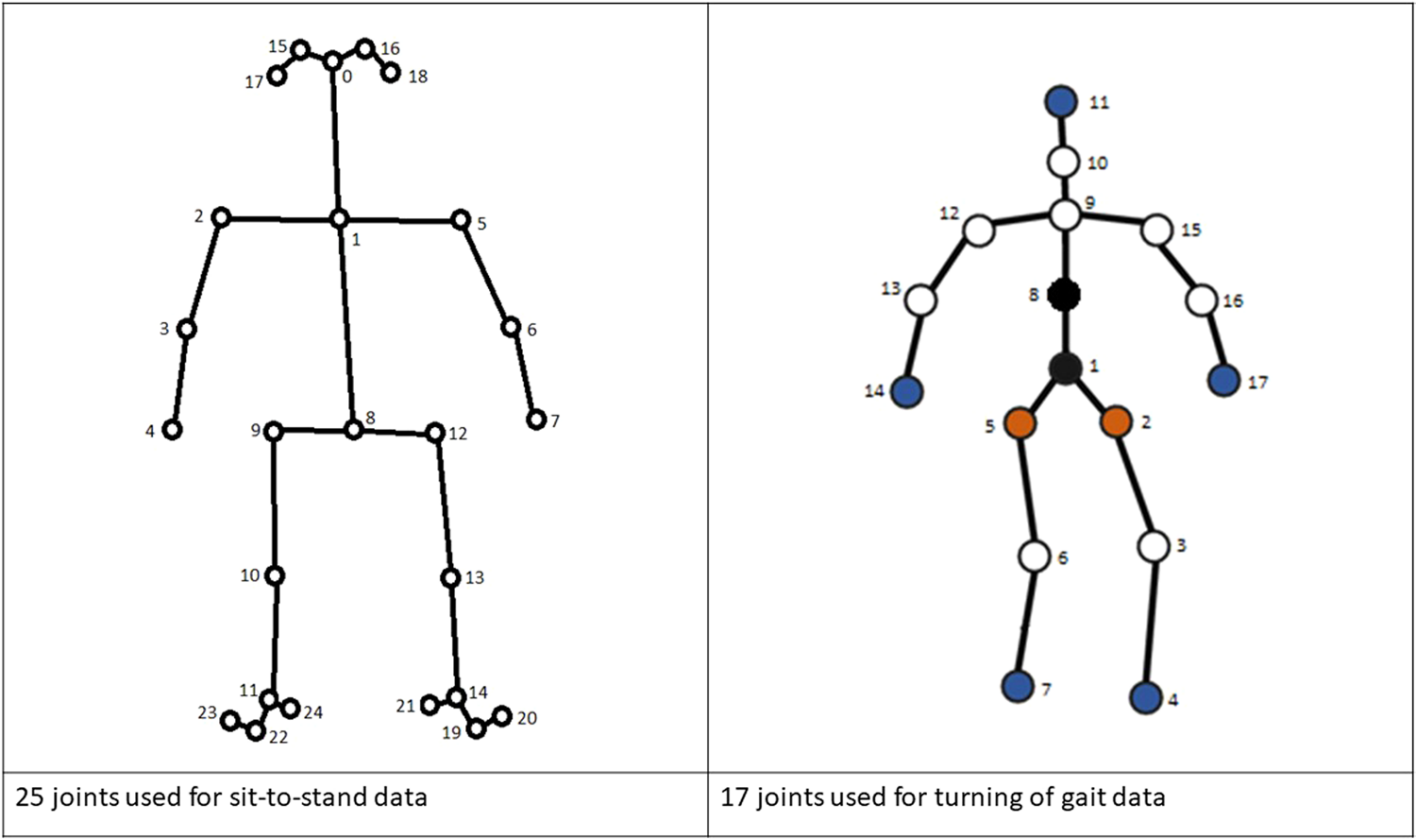
Layout of 2-dimensional skeleton joints used in sit-to-stand and turning.

Skeleton data was provided frame-by-frame, with each 2D (2-dimensional) data frame containing x and y coordinates for each of the skeleton joints. This dataset included 2D skeleton data for each STS and turning of gait clip. Illustration of the joints included in each case is in Fig. 4.

STS RGB videos were analysed using OpenPose software^62^ to detect human bodies and extract their skeleton joints. Most clips were 17 seconds long, with 2 seconds/2000 milliseconds included before the transition and a variable amount of data included afterwards to make up the total duration. For the avoidance of doubt, the STS transitions always start at 2.000 seconds; this information can be used to match the skeleton data with the accelerometer data. The extra added data may enable algorithms to be developed which identify STS transitions. However, because of various factors (e.g. subject disappearing from the frame), some of the clips can be shorter. Therefore, each frame was allocated a timestamp. The skeleton included 25 joints that can be divided into different parts of the body as shown in Fig. 4: head (0 nose, 15/16 eyes and 17/18 ears), trunk (1 neck and 8 mid hip), arms (2/5 shoulders, 3/6 elbows and 4/7 wrists), legs (9/12 hips, 10/13 knees, 11/14 ankles), and feet (19/22 big toes, 20/23 small toes and 21/24 heels). Each frame of STS skeleton data contained 2 (*x*, *y*) coordinates × 25 joints = 50 datapoints.

For turning of gait episodes, firstly RGB video clips were trimmed to contain the turning action with 6 frames of data/200 milliseconds included both before and after the action itself. These clips were then fed to a state-of-the-art 2D skeleton extraction model named High-resolution Net (HRNet)^63^. A pre-trained model already provided by the authors of the HRNet paper^63^ was used; the extracted 2D skeletons comprised 17 body joints shown in Fig. 4. The data was normalised as part of the methodology. Each frame of turning 2D skeleton data contained 2 (*x*, *y*) coordinates x 17 joints = 34 datapoints.

The open dataset^49^ skeleton data was coarsened (see Skeleton data re-idenfiability) to reduce identifiability, but all the joints described above (25 for STS, 17 for turning) were included in all skeleton data clips in both the open^49^ and controlled^50^ datasets.

#### 3D skeleton data

The turning RGB clips were also transformed to 3-dimensional (3D) human pose data. At first, having extracted 2D skeletal data from raw RGB clips^63^, these 2D skeletons were given as input to a strided transformer capable of producing a 3D pose estimation from a 2D source^64^. The method produced the corresponding 17 3D (*x*, *y*, z) coordinates of body joints. As with the 2D skeleton turning data described in the 2D skeleton data section, all 3D 17 (*x*, *y*, z) joints were also included in both open^49^ and controlled^50^ datasets and the data was normalised. Again, for the open dataset^49^, 3D data was coarsened to reduce the risk of re-identification.

#### Tri-axial accelerometry data

Tri-axial accelerometers were integrated into wrist worn wearables. A wearable was worn on each wrist by all participants throughout the study. The wearable devices were in-house designed and did not utilise gyroscope or magnetometer, a decision taken to maximise battery life. The accelerometer readings had a variable sampling rate of approximately 30 Hertz; the accelerometry records gave exact timestamps for each sample to enable accurate use. Accelerometry traces in three spatial directions (*x*, *y*, z) for each wearable were recorded at each time point. Accelerometry data was included in the controlled dataset^50^ for each discrete episode of STS or turning. Accelerometer data clips were trimmed to only include the action itself. Furthermore, for comparison purposes, the dataset also included start-to-end accelerometry readings for the other non-turning, non-STS activities (see list below) below that were labelled in the dataset. These had no extra annotations, unlike STS and turning.

Non-STS, non-turning activities:

- Descending stairs
- Going up stairs
- Leaning over from standing
- Semi-recumbent
- Sitting
- Standing still
- Standing with activity
- Stand-to-sit

The wearable locations (right or left wrist) and handedness were included in the controlled dataset^50^, alongside information about which side, if any, the PD participants experienced more severe symptoms.

### Human rater labels

The RGB videos were watched post-hoc by medical doctors who had undertaken training in the MDS-UPDRS rating score, including gait parameter evaluation. Various aspects of the participants’ movement were identified visually and quantified by the rater, producing “labels” (a set of parameter outcomes for each action)^65^. A widely-available software called ELAN^66^, commonly used for video analysis and labelling^67^, was used to synchronise and review up to 4 simultaneously-captured video files at a time. A pre-prepared label template was used by both clinician raters, selecting terms from controlled vocabularies presented in drop-down menus in order to reduce variability in label use. The actions of turning in gait and STS were chosen due to the frequency with which they occur (for turning, around 20 times an hour on average in free-living; for STS, 3–4 times an hour). The choice of what to label was also influenced by the position of the cameras available to the study researchers (as these were placed in downstairs communal rooms, some views were unavailable, such as, for example, a view of the whole staircase) and the knowledge that both turning and STS actions are often altered in mild-moderate PD^68,69^. Importantly, these actions are influenced by the core motor symptoms of bradykinesia and postural instability and therefore longitudinal repeated measurement of these actions shows potential as a measure of symptom, and therefore disease, progression in PD^70,71^.

Other actions were included to enable comparison with STS and turning (see details above). These are labelled from start-to-finish to the nearest millisecond. They are mostly self-explanatory (e.g. “Sitting”). “Leaning over from standing” indicates an action such as someone bending over to open a low cupboard. “Semi-recumbent” indicates a period where someone is seated with their legs raised on the sofa/footstool. “Standing with activity” includes any standing periods where the person was undertaking actions with their upper limbs but keeping their lower limbs relatively still (e.g. food preparation activities at the kitchen worktop).

The turning parameters labelled were: turning angle estimation (90° to 360° in 45° increments), number of turning steps (integers from 1 to 18), duration of turn (seconds:milliseconds), type of turn (pivot turn, step turn). 11 out of the 12 pairs (total 22 participants) had turning episodes evaluated.

A turning episode was defined as:

- Starting from the initiation of rotation of the pelvis, ending in completion of movement.
- Not a turn taken in a walking arc (e.g. walking around a table).
- Clearly visible from the video.

Turning angles were estimated visually by the human raters. Turns with angles of 45° were not included because the raters found it too challenging to reliably identify them and accurately quantify their duration. Turn angles were rounded to the nearest 45°, therefore for example all turns between 67.5° and 112.5° were included in the 90° angle dataset. There were turns where the person bent over from standing towards the end, or where the person started leaning down and turned as they stood up. Where the participants’ feet were visible in the video frame, the number of turning steps was counted. In terms of type of turn: a pivot turn was classified as a turn in which one or both feet swivel in place to achieve the turning movement; a step turn was classified as a turn achieved by three steps or more without pivoting. The episodes taken during “Clinical assessments” describe when the participants are undergoing the tests outlined in Clinical assessments. Otherwise, the data were defined as “free-living”.

Similarly, participants’ STS episodes were identified and labelled by non-clinician and clinician raters. Raters first provided the “whole episode duration” label which started when the participant made their first motion towards standing from a static sitting position and ended when the person was fully upright. A clinician rater then provided a second duration for all the STS episodes, the “final attempt duration” label in milliseconds, comprising their impression of the duration between the lowest point of the head (start) and when the person was fully upright/the maximum vertical position of the vertex of the head (end). The STS episode label was terminated before any walking steps were taken away from the upright position. The rater also noted: whether the person was dual-tasking - in this case whether they were carrying something in their hand(s) as they stood up; whether the attempt to STS was perceived as slow in their opinion; whether more than one attempt was taken for the transition; whether the arms of a chair (or other flat surface e.g. nearby table) were used to get up; or whether the person moved forward in the chair prior to their STS episode. Finally, a MDS-UPDRS score was allocated for each STS transition, using the scoring system set out in question 3.9 of this clinical rating scale tool:

- 0 = No problems. Able to arise quickly without hesitation.
- 1 = Arising is slower than normal; or may need more than one attempt, or may need to move forward in the chair to arise. No need to use the arms of the chair.
- 2 = Pushes self up from arms of chair without difficulty.
- 3 = Needs to push off, but tends to fall back; or may have to try more than one time using arms of chair, but can get up without help.
- 4 = Unable to arise without help.

If the person was positioned in the video frame in such as way as to occlude necessary additional visual information, where possible the STS episode was still labelled with “whole episode duration” and “final attempt duration”, but the MDS-UPDRS 3.9 score and additional information were not assigned. If it was not possible to discern the final attempt duration accurately (e.g. because of occlusion from another person), a result of 0 milliseconds was given.

### Combining the data

As mentioned in 2D skeleton data, the skeleton pose data has extra frames added before and after the discrete action. Knowing the timings of the action within the data will enable comparison of the different modalities for the same action:

- STS skeleton data: 2000 milliseconds are added before the STS transition and a variable duration of data is added after STS transition to make up to 17 seconds in total.
- Turning skeleton data: 6 frames/200 milliseconds are added before and after the turning action.
- Accelerometer data: discrete start-to-end clips are included.

### Data use case example: a computational approach to sit-to-stand speed and duration evaluation

#### Overview of approach

**Fig. 5:**
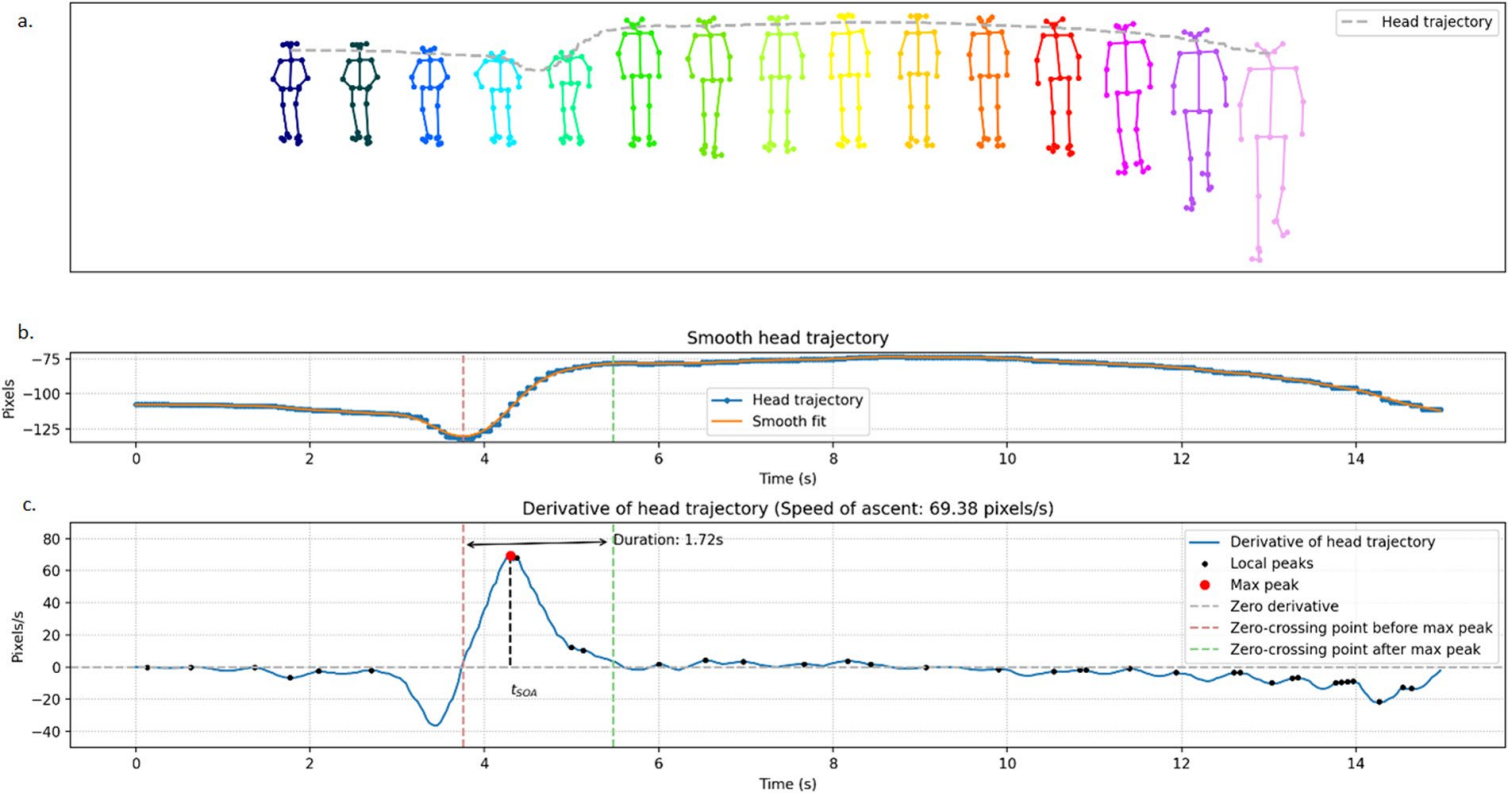
Illustration of the use-case automatic sit-to-stand speed and duration estimation approach.

Our group developed an approach to estimate STS speed and duration using the 2D skeleton data from this dataset. From the total of 25 joints, eight were chosen as the minimum number necessary to evaluate these parameters. The evolution in time of the head, or “head trajectory”, was estimated by averaging the vertical components of the five joints that compose the head (i.e. joints 0, 15, 16, 17 and 18) plus the three shoulder joints (i.e. joints 1, 2 and 5). The head trajectory was then used to estimate the STS parameters after being smoothed with a Savitzky-Golay filter^72^. This is a digital filter that can be applied to a set of digital datapoints for the purpose of smoothing the data (see part b. of Fig. 5). Then the speed of ascent (SOA) was evaluated similarly to our previous work^73^ - by finding at the maximum derivative of the head trajectory within each video clip. Figure 5 from the work by our group^74^ illustrates the process of STS speed quantification: visualisation a. shows the skeleton image sequence from a single STS transition; graph b. illustrates the head trajectory in pixels during the transition; graph c demonstrates the derivative of head trajectory which is then used to calculate the speed (in pixels/second) and duration of ascent (in seconds). Secondly, the STS duration was estimated - in this case this meant the time elapsed between when the head was at its lowest and highest points in the frame. This process uses a moment in time identified by the STS speed quantification approach, namely tSOA (time identified through the SOA approach). Once tSOA is found, the closest local minimum (when the person’s head is at its lowest point in the frame) before tSOA and the closest local maximum (highest point someone reaches in frame after standing up) after tSOA are identified. Finally, the time elapsed between these two points is calculated to estimate STS duration. This is illustrated in part c of Fig. 5 by looking at the points where the derivative of the head trajectory crosses the zero line.

An important step in measuring STS parameters is their conversion to physical measurements. While the STS duration can be easily converted from frames to seconds (s) knowing the frame-rate of the camera, the conversion to STS speed requires additional processing. In fact, skeleton coordinates are measured in pixels, therefore the STS speed is expressed in pixels/second (pixels/s). These values must be converted into physical quantities (metres per second) before different measurements can be compared, otherwise the STS speed will depend on the distance of the subject from the camera. To override this issue without having to perform lengthy calibration procedures, we normalise the STS speed by the height of the skeleton, in pixels^75,76^, at the moment they complete the transition (i.e. zero-crossing point after max peak). This value, multiplied by the height of the subject in metres, allows us to estimate the speed of each subject in metres per second.

It is important to note that the STS duration detected by this use-case automatic approach only considers the time elapsed from when the person starts moving upwards until the movement is terminated; therefore, in case the participants require several attempts at standing up, only the last attempt will be timed. While this behaviour can create some discrepancies with the STS duration times measured by clinicians in a lab setting (which instead start timing from when the subject first moves towards standing up), this helps improve robustness and consistency of the algorithm in a free-living environment.

Although the analysis performed in this work used the human rater label timestamps to identify STS episodes, the entire process (including STS detection) can be fully automated, as we showed in our previous work, to detect STS transitions in the real-world^73^.

#### Filter size impact

The impact of the filter size for the Savitzky-Golay filter was also studied for our use case (NB the data in the REMAP dataset itself is raw data). This is defined by the window size and the polynomial order used to fit the head trajectory data and estimate the derivatives. In our experiment, we noticed that a smaller window size is beneficial for estimating STS speed but produces higher errors in the STS duration, while a larger window size has the opposite effect. This phenomenon is to be expected since larger window sizes increase smoothing and promote a reduction of false positives in the peaks used to estimate the STS duration. However, higher smoothing also means reduced gradients, which has a negative impact on the estimation of the STS speed. A compromise between the two measurements must therefore be made to obtain optimal STS parameters: we chose a window size of 11 and polynomial order of 1.

### Skeleton data re-identifiability

High resolution RGB colour video, especially (but not only) when it shows the person’s face, is clearly re-identifiable personal data. Producing skeleton joint markers from RGB data reduces, but does not remove fully, information about the face, clothes and accessories, and body habitus. However, skeleton pose data also retains important information about someone’s posture, gait and habitual poses (e.g. if someone had a habit of standing in a certain way that was particularly identifiable). It has been shown that skeleton data can reveal gender^77^, age to a 10-year range^78^ and, if full gait cycles are shown, be re-identifiable to the person in the video to a high degree of accuracy (>80%)^79,80^. Therefore, before this kind of data is released openly, careful consideration must be given to minimising re-idenfiability whilst retaining the useful content of the data. The most discriminating joints for re-identification through gait data are hands (e.g. giving information about arm swing) and feet (e.g. with stride length and duration)^79^. These joints in our data are potentially interesting to researchers as they contain information about how someone transitions from seated to standing (e.g. if they are using the arms of the chair) and about turning in gait (e.g. how many steps they take in a turn), so we preferred to include all the skeleton joints to enhance dataset utility.

However, unpublished work studying different aspects of detailed gait data has explored different ways of changing the data to reduce gait and identity recognition^81^. Using one of their approaches (which they tested on data with higher resolution), we coarsened the skeleton data in the open dataset^49^ by mildly perturbing the joint coordinates to reduce the risk of person re-identification. We have also applied other methods to reduce re-identifiability from the dataset as a whole, described below in Reducing re-identifiability: methods applied to the data.

### Trial registration

As this was an observational study it was not registered with a trials database but instead was registered with the University of Bristol (the study sponsor), reference number 2018–4247 and on the research database of North Bristol NHS Trust, study reference number 4475.

## Data Records

Due to the fact that much of this data is personal, to protect privacy and adhere to the written consent gained from each participant, we have taken the decision to divide our dataset into open and controlled components. Both components are found in the University of Bristol’s Research Data Repository (data.bris).

The controlled dataset^50^ contains pseudonymous data including the full skeleton data for all STS and turning episodes. It also contains the bilateral wrist-worn accelerometry data for these episodes from all participants, along with accelerometry data for non-turning, non-STS action episodes. The reason the accelerometry data is in the controlled dataset is because the proven re-identifiability risk of accelerometry^82^ contravenes the study consent conditions relating to data sharing. We include individual-level demographic and clinical rating scale score outcomes given in ranges. The controlled dataset^50^ is available on an application basis.

The open dataset^49^ contains data which is anonymous, either because it is given at cohort-level (e.g. demographic data), because the labels contain non-personally identifiable content (PD vs C status) or because the data has been processed in such a way (see Reducing re-identifiability: methods applied to the data) to render it extremely unlikely to be re-identifiable.

### Sensor dataset

Nomenclature for all individual files/folders containing the final sensor data for an individual mobility action episode: Pt[unique ID number]_[PD/C]_n_[turn/STS/NonTurnNonSTS episode number].

- Pt = participant.
- ID = identification.
- n = turn/STS/NonTurnNonST episode number, as defined in the human rater label spreadsheets.

### Human rater label dataset

The human rater label dataset for STS and turning is included in both the open dataset^49^ and the controlled dataset^50^. Human rater labels are also included for the non-turning, non-STS accelerometer data in the controlled dataset^50^. The information included in the human rater label dataset is outlined in Table 3.

#### Turning of gait

The turning of gait human rater labels are contained in the spreadsheet named turning_human_labels.xls. Each row in this spreadsheet comprises one turning of gait episode from one participant. Column A allocates a unique number to each STS episode. This unique number is then linked to data from three sensor data modalities: the 2D skeleton data, the 3D skeleton data and the accelerometer data from each wrist of the participant (only in the controlled dataset^50^).

#### Sit-to-stand

The STS human rater labels are contained in the spreadsheet named SitToStand_human_labels.xls. Each row comprises one STS episode from one participant. Column A allocates a unique number to each STS episode. This number then corresponds to the sensor data files from two modalities: 2D skeleton and accelerometry (only in the controlled dataset^50^).

#### Non-turning, non-STS

All non-turning, non-STS actions are included in the folder called “NonTurnNonSTS”. This contains a master spreadsheet with human rater label information about all actions named NonTurnNonSTS_human_labels.xlsx. Also within this folder are sub-folders with the data and human rater label spreadsheets from each individual action (DescendingStairs, Sitting etc). These spreadsheets have the same layout and column title structure as those for turning and STS.

### Open dataset

In addition to the human rater label dataset described in Table 3, the open dataset^49^ contains 2D/3D skeleton pose data, which is effectively anonymised^83^, whilst maintaining its potential for use to evaluate the mobility-related activities of interest. The sensor data in the open dataset^49^ is outlined in Table 5. Other information in the open dataset includes the code developed by our team for the use case of STS speed evaluation using our skeleton data. The open dataset^49^ may be accessed via this 10.5523/bris.21h9f9e30v9cl2fapjggz4q1x7.

**Table 5:** Sensor data from cameras or wearables: contents of open dataset.

Data type	Sensor	What is included	Sit-to-stand	Turning
2D skeleton data	Camera	All skeleton joints for each frame of the clip: 2 joint coordinates (x and y) per joint per frame.	×	×
3D skeleton data	Camera	All skeleton joints for each frame of the clip: 3 joint coordinates (x, y and z) per joint per frame.		×

### Controlled dataset

The controlled dataset, available through an application process described in^50^, comprises the sensor data listed in Table 6.

**Table 6:** Sensor data from cameras or wearables: contents of controlled dataset.

Data type	Sensor	What is included	Sit-to-stand	Turning	Non-turning, non-sit-to-stand
2D skeleton data	Camera	All skeleton joints for each frame of the clip. 2 joint coordinates (x and y) per joint per frame.	×	×	
3D skeleton data	Camera	All skeleton joints for each frame of the clip: 3 joint coordinates (x, y and z) per joint per frame.		×	
Tri-axial accelerometry	Wrist-worn werable	Accelerometry traces from each of the two wearables worn by each participant for all clips.	×	×	×

Additional data in this dataset includes:

- Demographic data: age (in 5-year range), gender, years since diagnosis (in 3-year range), levodopa equivalent daily dose (to nearest 100 mg), whether or not they have deep brain stimulators.
- Clinical rating scale and questionnaire scores (with ranges):
  *Motor* MDS-UPDRS total and part III scores, postural instability gait difficulties sub-score of MDS-UPDRS and TUG-test times, specifying the “on” and “off” medication status;
  *Non-motor* NDS-UPDRS non-motor part I, Parkinson’s Disease Sleep Scale score, Non-motor Scale Score, Rapid Eye Movement Sleep Disorder Screening Questionnaire score, Parkinson’s Disease Quality of Life Questionnaire-39 total score and Mobility/Activities of daily living subscores.
- Information on wearable position (participant, left/right wrist), left or right-handedness for all participants and PD symptom side dominance (if asymmetrical) for PD participants.

N.B. The levodopa equivalent daily dose is a conversion of a person’s dopaminergic parkinsonian medications to generate a total L-dopa equivalent daily dose, calculated as a sum of each medication.

The controlled dataset^50^ may be accessed via this 10.5523/bris.2o94rzjooyzf42w850dqg0spfh.

### Reducing re-identifiability: methods applied to the data

#### Methods applied to data from both open and controlled sections

The steps that have been taken to reduce identifiability of all data include:

- Removing the timestamps from the data so that the date and time of study participation is hidden.
- The use of fresh unique identifiers for the participants so that their data cannot be traced to previously published works.
- The computer vision (skeleton pose) data has a temporal jitter introduced due to the subtly variable frame rate of data collection.
- All turning of gait data clips are trimmed to include the relevant action only, with no full “walking straight-ahead” gait cycles. Data clips are therefore short (restricted to a few short seconds only), therefore reducing the likelihood of biometric data leakage from this data. All STS data clips are only 17 seconds in duration, and therefore these are unlikely to contain any easily visible, side-on-to-camera, natural straight-ahead gait cycles.
- All turning data is normalised (the pre-processing of data to appear similar across all records) through the process of generating the 2D and 3D skeletons.
- The STS data is not normalised, but it is in the pixel space, not in the 3D world coordinate space. This means that there is no way of knowing the participants’ heights, reducing the re-identifiability risk from the STS skeleton data.

#### Methods used for open dataset only

The additional methods used to reduce re-identifiability in the open dataset^49^ include:

- Coarsening both the STS and turning data as described in Skeleton data re-identifiability^81^.
- Only including cohort level demographic and clinical rating scale score information. The only specific clinical information given at an individual level is the diagnosis (or not) of PD and presence (or not) of a deep brain stimulator.

Using the methods described above, guided by the current literature on re-identifiability of skeleton data, we are confident that the open data is effectively anonymous. This open dataset^49^ is designed to be shared as widely as possible, in line with our group’s motivations for the data to be used for the greatest benefit possible, but respecting the need to keep the participants’ identities anonymous.

## Technical Validation

### Participant pseudonymisation

Participants were each allocated a unique study ID, which was used throughout the study. The real identifiers of the participants were hidden from the research team except on a need-to-know basis, such as technical support, or direct meetings between the research team and participant for purposes such as consenting or data collection. Then, for this dataset publication, new unique identifiers were assigned to each participant.

### Human label inter-rater agreement

#### Turning of gait labels

Two clinicians annotated 50% of the turns each. Around 50% of the total number of annotations were cross-checked (randomly selecting 6 pairs from 11) by both clinician annotators, blinding the cross-checking clinician to the turning annotations produced by the other. Cohen’s Kappa^84^ statistic was calculated to evaluate inter-rater reliability.

The two clinician raters had an almost perfect^85^ inter-rater agreement for turning angle (Cohen’s kappa = 0.96) and number of turning steps (Cohen’s kappa = 0.97) annotations. The agreement rate was 97% for turn duration. Any discrepancies were recorded, discussed, and resolved by the clinician raters, and with a final review by a movement disorders specialist.

#### Sit-to-stand labels

A number of different non-clinician and clinician raters identified and labelled (start-to-end, producing the “whole episode duration” labels) the STS episodes from the RGB data. All of the STS episodes were then re-watched by a single clinician rater (a neurology specialty registrar who had completed training in MDS-UPDRS scoring, including evaluation of STS and its subcomponents). This rater checked and, where they felt necessary (which occurred for 5% of the labels), adjusted the whole episode duration labels. They added a further “final attempt duration” label as discussed in Human rater labels. 10% of the STS episodes were chosen to evaluate intra-rater reliability, with this sub-set scored several months after the first rating by themselves. The intra-rater agreement on labels was 98% for “final attempt duration” and 98% for the MDS-UPDRS part 3.9 score. Cohen’s kappa^84^ was run to determine the reliability of the single rater rating the same labels twice, while correcting for how often the rater may agree with themselves by chance. The Cohen’s kappa was 0.97 for MDS-UPDRS part 3.9 scores, showing an almost perfect level of agreement^85^.

## Usage Notes

### Restrictions for controlled dataset

Due to the novel nature and collection method for these data, governance structures have been put in place in order to respect the balance between the desire of participants to share their data and the respect for privacy of those participants. Researchers who are interested in accessing these data need to complete the following steps: submit an Intended Data Use statement and agree to the Data Use Agreement associated with each data source (see Digital Object Identifiers (DOIs) for each data source). The overarching conditions in the Data Use Agreement are as follows:

- You agree to sign and abide by the conditions within the Research Data Access Agreement For Controlled Data provided by the University of Bristol upon application for this controlled dataset.
- You agree to abide by the guiding principles for responsible research use and data handling as described in UKRI research integrity policy documents https://www.ukri.org/about-us/policies-standards-and-data/good-research-resource-hub/research-integrity/.
- You confirm that you will not attempt to re-identify research participants for any reason, including for re-identification theory research.
- You commit to keeping these data confidential and secure.
- You understand that these data may not be used to re-contact research participants.
- You agree to report any misuse or data release, intentional or inadvertent to the data controllers within 5 business days by emailing data-protection@bristol.ac.uk.
- Data users are strongly encouraged to cite this Data Descriptor, the associated study protocol^86^ and one or both of the open^49^ and controlled^50^ datasets, via formal citations in your article reference lists, including the DOIs, to allow for formal accreditation of the data creators. Please see the dataset references^49,50^ in this article for the format to use.

### Ideas of experiments you could do on this dataset

Ideas for future experiments using the REMAP dataset include designing an approach to automatically classify PD or control status based on how the person does a STS transition, or turns in gait. One could explore how to classify whether someone is freely living or undertaking a clinical assessment when they are performing the actions in this dataset, or detect when someone is dual tasking when they stand up from sitting down. Different approaches could be explored towards calculating the turn angle, duration or number of turning steps taken in each clip, or calculating the duration of the STS transition.

## Data Availability

The code for 2D and 3D skeleton pose data generation and the use-case code evaluating STS speed is available here: https://github.com/ale152/SitToStandPD.
